# A Polymeric Platform for Whispering Gallery Modes Silica Microresonators Coupling

**DOI:** 10.3390/polym18202581

**Published:** 2026-10-20

**Authors:** Gianluca Persichetti, Vito Coviello, Daniele Farnesi, Gabriele Frigenti, Stefano Pelli, Genni Testa, Gualtiero Nunzi Conti, Romeo Bernini

**Affiliations:** 1 Institute for Electromagnetic Sensing of the Environment (IREA), National Research Council of Italy (CNR), 80124 Napoli, Italy; persichetti.g@irea.cnr.it (G.P.); coviello.v@irea.cnr.it (V.C.); testa.g@irea.cnr.it (G.T.); 2 Institute of Applied Physics “Nello Carrara” (IFAC), National Research Council of Italy (CNR), 50019 Sesto Fiorentino, Italy; d.farnesi@ifac.cnr.it (D.F.); g.frigenti@ifac.cnr.it (G.F.); s.pelli@ifac.cnr.it (S.P.); g.nunziconti@ifac.cnr.it (G.N.C.)

**Keywords:** WGM resonators, polymer waveguides, coupling efficiency, grating couplers

## Abstract

A polymeric platform for high-performance coupling to whispering gallery modes of silica microresonators is proposed and validated. The system is based on a low-refractive-index ridge waveguide fabricated with widely used SU8 and PDMS optical polymers. The waveguide is integrated with grating couplers for a simple fiber-to-chip connection. The coupling capabilities of the proposed platform were characterized using microsphere and microbubble resonators of different sizes. The experimental results demonstrate coupling efficiency in excess of 99% with microsphere and microbubble resonators having intrinsic quality factors in excess of 10^7^ and 10^6^, respectively. This performance is comparable with conventional fiber taper couplers and enables simple on-chip packaging of silica microresonators.

## 1. Introduction

Optical silica microresonators represent a powerful tool in several applications like sensing, lasing, and nonlinear optics [1,2,3,4]. Microsphere, microbottle and microbubble resonators, supporting whispering gallery modes (WGMs), are able to achieve very high quality factor (Q) with small mode volume, enabling exceptional optical properties and allowing strong light-matter interaction. In particular, fused silica microresonators offer the advantage of combining high performance with a simple and low-cost fabrication process based on heat melting.

However, one of the main challenges that must be addressed toward the effective application of optical microresonators is their integration with an efficient and reliable optical coupling mechanism [5]. Presently, the optical fiber taper represents the most widely used coupling approach due to high efficiency and simplicity, but tapered fibers are mechanically fragile and very sensitive to environmental factors. In recent years, a great effort has been devoted to the development of new coupling approaches based on integrated planar waveguides. To achieve high coupling efficiency (CE), i.e., a highly efficient transfer of energy from an external light source to the resonator, it is essential to satisfy the phase-matching condition [6]. This implies that the effective refractive index (n_eff_) of both the waveguide modes and the resonator WGMs must be in close proximity to one another. The phase-matching condition of a planar waveguide with a silica resonator (n_silica_ = 1.44) can be achieved both with low refractive index waveguide materials and with advanced waveguide geometries like nanowires, photonic crystals, and metamaterials. A material-based approach has been successfully proposed employing fluorinated polymer (Q = 3.4 × 10^6^, CE = 91%) [7]. Femtosecond-written surface waveguides in a silica substrate have been employed as a coupler to a microbubble resonator (Q ≈ 10^4^, CE ≈ 6.5%) [8]. Integration of microbottle resonators with silicon nitride waveguides has been realized with moderate performances (Q ≈ 6 × 10^4^) [9]. Advanced geometrically engineered silicon waveguides represent an alternative approach to the coupling problem that, up to now, has achieved the best performance. Stripline pedestal anti-resonant reflecting optical waveguide (SPARROW) was able to achieve a CE of 95% and a Q-value of 5 × 10^7^ for a microsphere of diameter 220 µm [10]. However, these waveguides are highly wavelength-dependent because they rely on the resonant nature of reflections from multiple dielectric layers in the cladding. A silica WGM microresonator has been directly coupled to a silicon chip by employing a silicon photonic crystal waveguide with a Q = 1.34 × 10^6^ and a CE up to 99.5% [11]. Farnesi et al. realize Si waveguides based on subwavelength metamaterial engineering, enabling a CE = 99% with silica microspheres with a Q up to 2 × 10^7^ [12]. However, these approaches require rather complex fabrication processes or very high-resolution lithography.

In this work, we propose and validate a new polymeric integrated optical platform that allows for efficient coupling to high-Q silica optical microresonators like microspheres and microbubbles. The photonic chip is based on ridge waveguides realized with standard polymers like SU8 and Polydimethylsiloxane (PDMS), widely used in integrated optics microfabrication [13,14]. The adoption of these materials allows us to realize low refractive index waveguides and achieve good phase-matching conditions with silica microresonators via a very simple fabrication process. The polymer waveguides were also integrated with grating couplers, which avoided cleaving and polishing procedures, thereby simplifying the fiber-to-chip connection and unlocking the entire 2D chip area for effective device integration. The proposed platform enables coupling performance comparable to a tapered optical fiber coupler, with the advantages of planar technology, allowing simple and effective packaging of silica microresonators [8,15].

## 2. Materials and Methods

### 2.1. Design and Fabrication of Integrated Polymeric Platform

**Figure 1:**
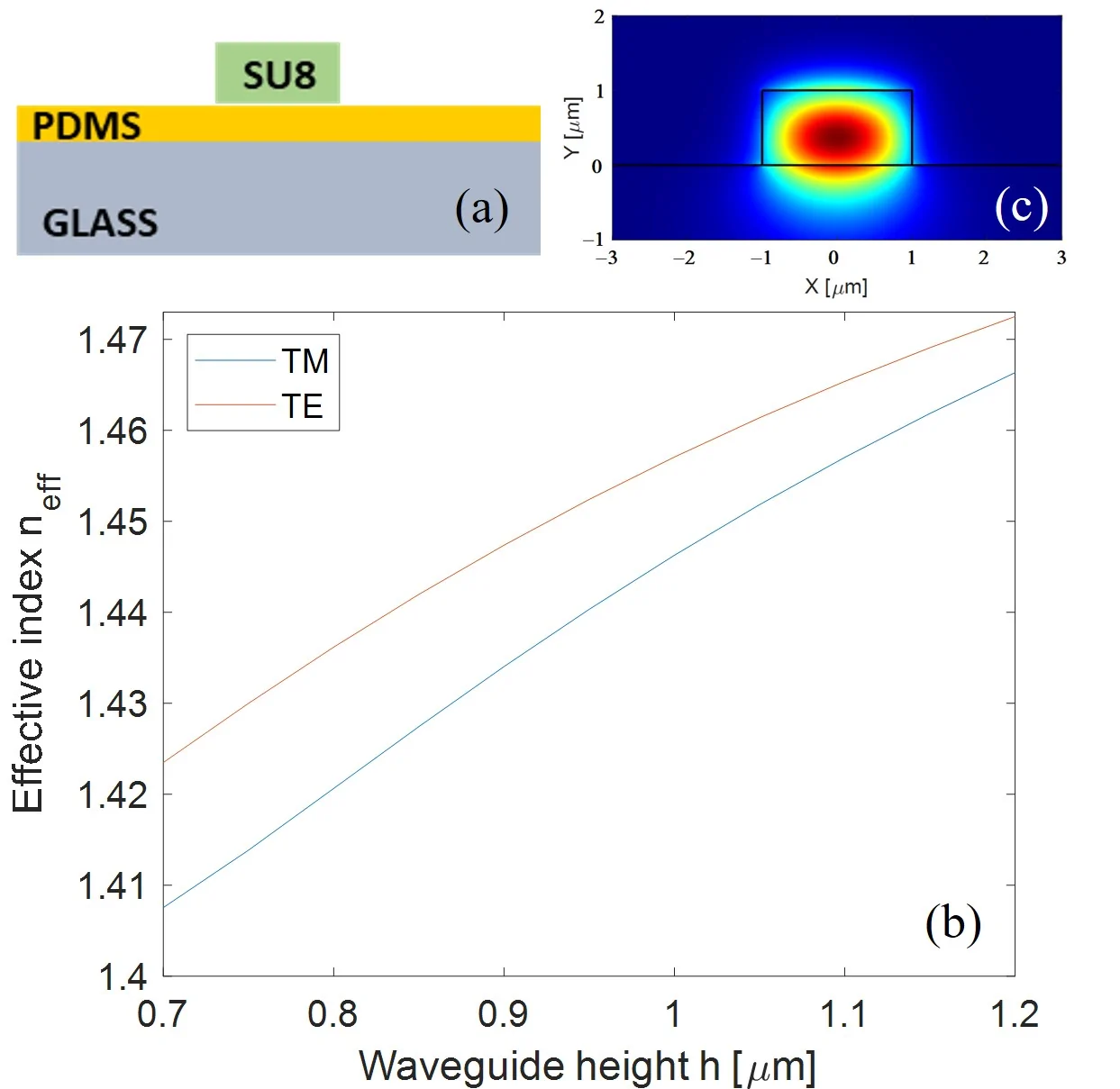
(**a**) Section of a ridge polymeric waveguide on a glass substrate, (**b**) TE and TM effective refractive indices of 2 µm wide ridge waveguide as a function of its height at a wavelength of 1550 nm, (**c**) TM mode field distribution of a 1 µm high and 2 µm wide waveguide at a wavelength of 1550 nm.

The schematic of the planar waveguide proposed for coupling with silica WGM resonators is depicted in Figure 1a. In this approach, the ridge waveguide is made with SU8 core on a PDMS layer. The waveguide substrate consists of borosilicate glass. Numerical simulations using the finite element method (FEM, Comsol 5.6, Electromagnetic Waves, Frequency Domain) were performed to define the waveguide geometry, ensuring single-mode propagation conditions and optimal phase matching with the WGMs of the resonators. The refractive index of PDMS and SU8 at 1550 nm were set to n_PDMS_ = 1.4050 and n_SU8_ = 1.5750, respectively [13]. The simulations showed that for a 2 µm wide waveguide, varying the waveguide height *h* in a range between 0.7 µm and 1.2 µm allows tuning of the waveguide effective refractive index of TM and TE modes approximately from 1.40 and 1.47 (Figure 1b). Figure 1b shows that TE operation requires a smaller waveguide height with respect to the TM to achieve the same effective index, which increases the risk of higher overall losses [16]. For this reason, we have focused our attention on TM polarization only. In Figure 1c, the TM mode field distribution of a 1 µm high and 2 µm wide waveguide at a wavelength of 1550 nm is depicted.

**Figure 2:**
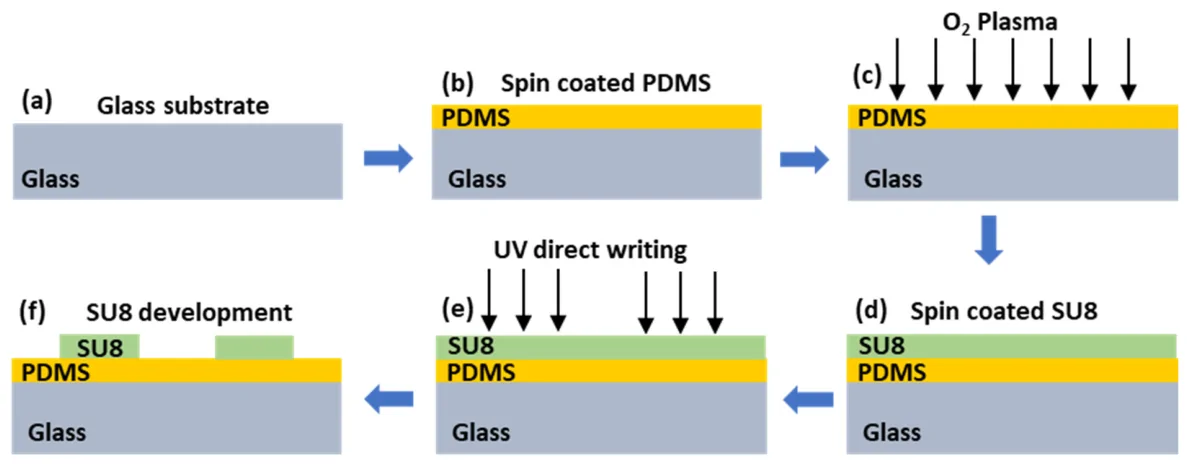
SU8 on PDMS waveguide fabrication process. (**a**) Substrate cleaning. (**b**) PDMS spin-coating. (**c**) Oxigen plasma treatment. (**d**) SU spin-coating. (**e**) UV laser direct writing lithography. (**f**) SU8 development.

The fabrication process flow is reported in Figure 2. A thick (30–40 μm) PDMS layer was obtained by mixing Silgard 184 (DOW, Midland, MI, USA) and a curing agent in a ratio of 1:10. PDMS was spin-coated (2000 rpm) on glass substrates (round microscope coverslips with a diameter of 3 cm) (Figure 2b). The microscope coverslips were preliminarily cleaned with acetone and dried on a hotplate at 70 °C for 30 min. After spin-coating process, the samples were placed on a hot plate and cured at 80 °C for 150 min. This PDMS layer was exposed to oxygen (O_2_) plasma (oxygen flow of 500 sccm at a pressure of about 5 × 10^−4^ bar for 60 s with a power of 40 W) to activate the surfaces of the PDMS and improve adhesion with SU8 (Figure 2c). SU8 2002 (solid content 29.00%) was diluted with SU8 Thinner to obtain a SU8 formulation with 25.33% solids content. The resulting mixture was spin-coated (3000 rpm) onto the PDMS layer right after the preparation of the PDMS surfaces (Figure 2d). The SU8 layer was soft-baked on a hot plate at 80 °C for 15 min and then exposed to ultraviolet (UV) light (375 nm, 11.2 mW, write speed 0.75 mm^2^/min) using a laser direct writing lithographic system (μPG 101, Heidelberg Instruments Mikrotechnik GmbH, Heidelberg, Germany) to define the waveguide geometry (Figure 2e). The UV-exposed SU8 layer was heated again at 80 °C for 150 min for the post-exposure bake process, cooled to room temperature, and immersed in SU8 developer bath composed of propylene-glycol-monoether-acetate (PGMEA) to dissolve the uncrosslinked SU8 (Figure 2f). The resulting waveguide height was approximately h ≈ 0.96 µm, as measured using an optical profilometer.

The refractive index of the deposited layers was determined via m-line spectroscopy measurements at two different wavelengths, obtaining the results shown in Table 1, where experimental errors are estimated as standard deviation of repeated measurements. These values are in excellent agreement with those used in the simulations.

**Figure 3:**
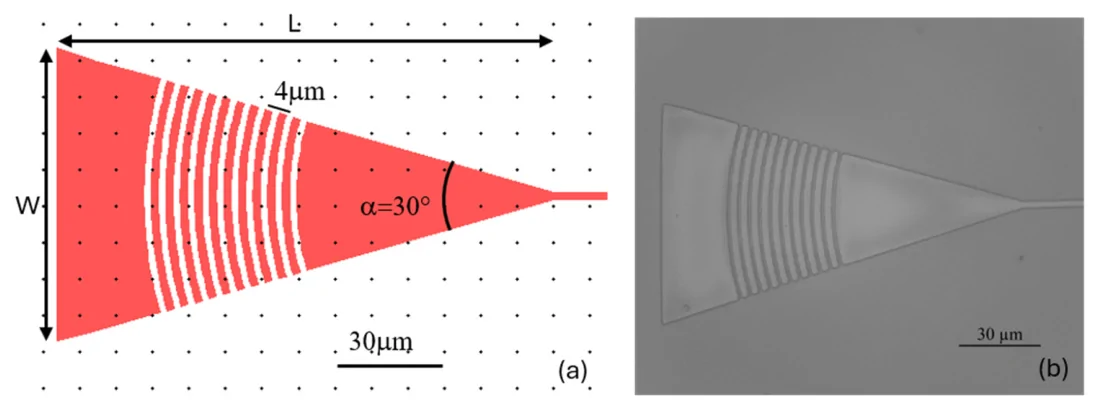
(**a**) Characteristics of the grating coupler. (**b**) Picture of the grating coupler.

The coupling of light into and out of the waveguide was performed using two grating couplers. The gratings are located at the ends of the waveguide and have the characteristics shown in Figure 3a. A fully etched grating (h ≈ 0.96 µm) configuration was selected to maximize refractive index contrast and simplify the fabrication process, enabling the simultaneous creation of the waveguide and the grating couplers. The grating has a focusing geometry with grating lines (wide W = 74 µm, length L = 138 µm) with an opening angle of α  =  30°, a 4 µm grating period and a duty cycle of 0.5. The total number of periods is 10. The coupler is tapered down to the waveguide width of 2 μm with a compact geometrical footprint. This strongly tapered design avoids optical losses in long adiabatic tapers of TM-waveguides [17]. The fiber-to-chip insertion loss is approximately 20 dB. The maximum coupling value is about 1% around 1558 nm with 3 dB bandwidth of 54 nm covering the full telecom C-band. An image of the fabricated grating coupler is shown in Figure 3b.

### 2.2. Fabrication of Silica Microresonators

Microsphere and microbubble resonators were fabricated by melting technique based on arc discharge. Microsphere resonators (MSRs) were fabricated directly from the tip of an optical fiber (SMF-28), while microbubble resonators (MBRs) were fabricated from commercial silica capillaries (Postnova Analytics GmbH, Landsberg am Lech, Germany) with an external diameter of 150 μm [18].

### 2.3. Experimental Set-Up

**Figure 4:**
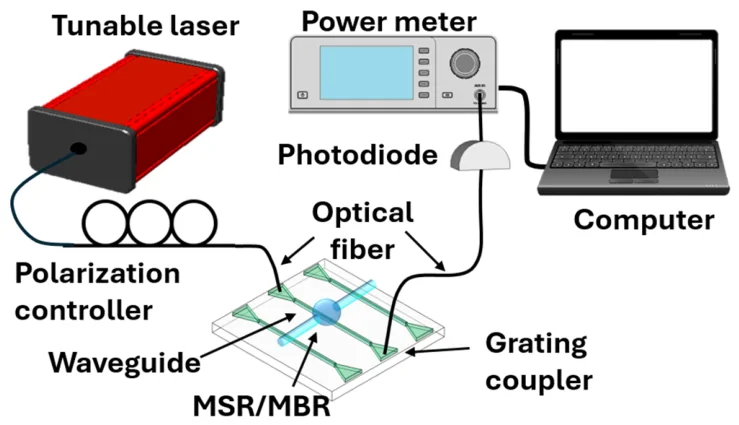
Experimental setup.

The experimental characterization was performed with an external-cavity tunable laser diode (Santec TLC 550, Tokyo, Japan) with an emission wavelength range of 1500–1630 nm and a linewidth of 200 kHz. The linearly polarized laser light was coupled to a single-mode fiber aligned to the input grating. TM polarization was set by a polarization controller. The optical power transmitted through the output grating was collected by a single-mode fiber connected to a high-speed optical power meter (Santec MPM-212, Tokyo, Japan), synchronized with the tunable laser, enabling real-time acquisition of the transmission spectrum as a function of wavelength. A 3D micro-positioning stage and an optical microscope were used to precisely align the resonator to the waveguide. During the alignment procedure, the transmission spectrum was monitored continuously to maximize the CE. A schematic of the experimental setup is shown in Figure 4.

## 3. Results

### 3.1. Microsphere Resonator

**Figure 5:**
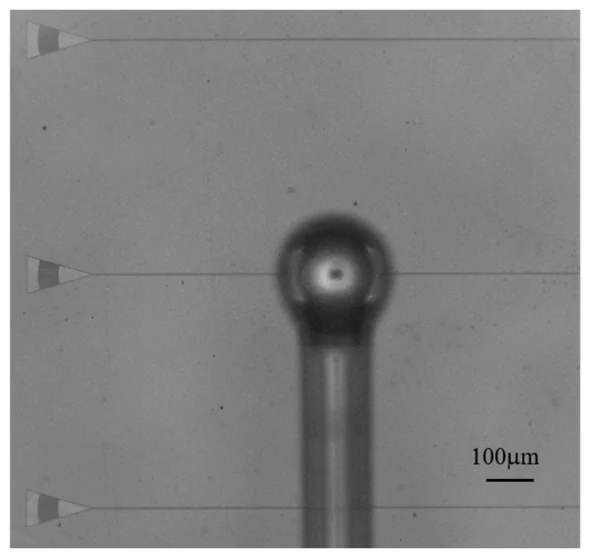
Microscope image of a silica microsphere positioned on the platform chip with an array of polymer waveguides.

The performance of the system was evaluated with a fused silica microsphere resonator with a diameter of 250 µm. Figure 5 shows a picture of the MSR positioned over the chip containing a set of polymer waveguides.

**Figure 6:**
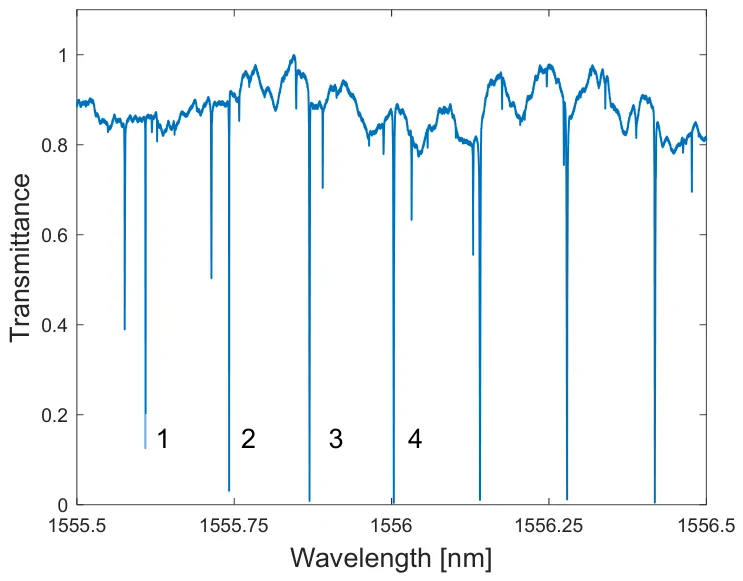
Transmission spectrum of the polymeric platform coupled to a silica microsphere resonator with a diameter of 250 µm. Q = 1.19 ± 0.01 × 10^7^ with CE = 85.5 ± 0.6% (resonance n.1), Q = 5.96 ± 0.14 × 10^6^ with CE = 96.6 ± 1.0% (resonance n.2), Q = 3.35 ± 0.03 × 10^6^ with CE = 99.1 ± 0.4% (resonance n.3), Q = 2.15 ± 0.02 × 10^6^ with CE = 99.6 ± 0.6% (resonance n.4)

**Figure 7:**
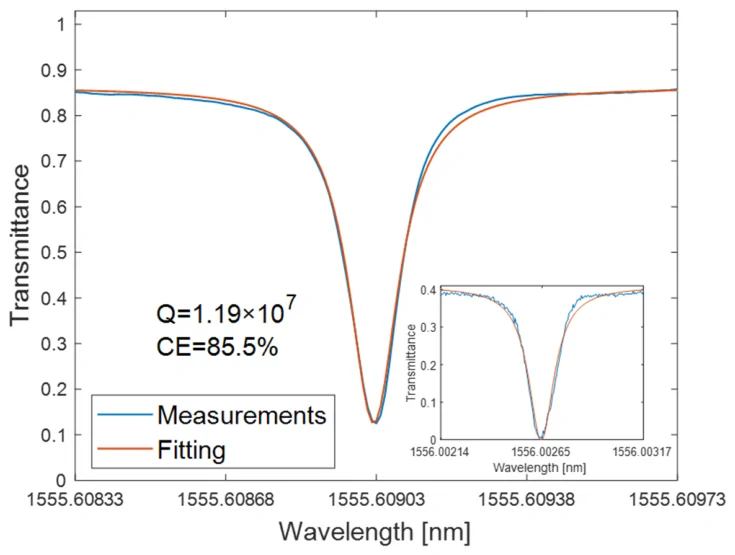
WGM resonance (n.1 in Figure 6) and its Lorentzian fit around λ = 1555.60 nm. The inset shows a similar WGM resonance measured using a fiber taper.

**Figure 8:**
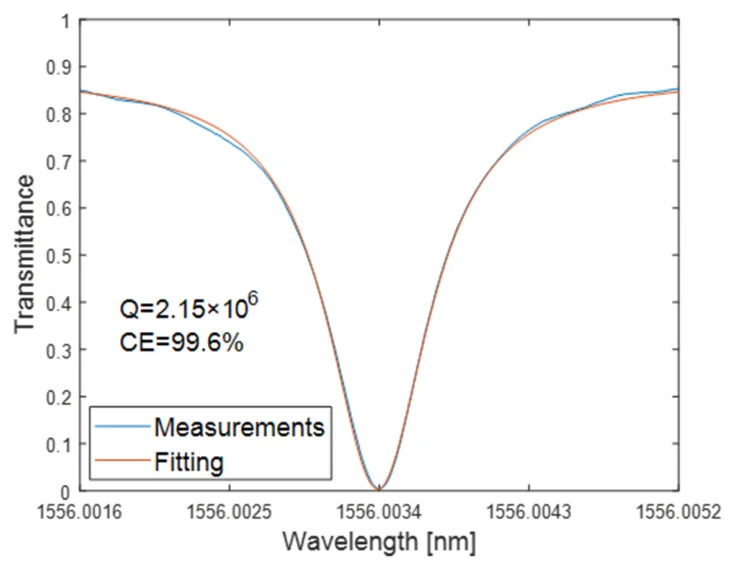
WGM resonance (n.4 in Figure 6) and its Lorentzian fit around λ = 1556.00 nm.

The transmission spectrum of a polymer waveguide coupled to the MSR is shown in Figure 6. The ripple-like fluctuations over the baseline are attributed to the back-reflection from the grating couplers due to the residual effective index mismatch between the waveguide and the grating region [19]. The device resonances were characterized in terms of loaded quality factor, defined as Q = λ_0_/Δλ, where λ_0_ is the resonance wavelength and Δλ is the full width at half maximum of the resonance depth, and coupling efficiency, defined as CE = (T_0_ − T_min_)/T_0_, where T_0_ is the off-resonance transmission and T_min_ is the minimum transmission or resonance depth [20]. It is worth recalling that the inverse of the loaded Q factor is the sum of the inverses of the intrinsic Q factor (governed by the intrinsic resonator losses) and the loading (or coupling) Q factor (governed by the coupling from the waveguide to the resonator). Consequently, the loaded Q factor is always lower than the intrinsic Q factor, establishing a lower bound for its value. The measured maximum quality factor, with the standard error (or uncertainties, which were estimated as 95% confidence intervals derived from the fit residuals), is Q = 1.19 ± 0.01 × 10^7^ with CE = 85.5 ± 0.6% (resonance 1). In Figure 7, a detailed view of this resonance fitted with a Lorentzian curve is depicted. Resonances 2, 3 and 4 exhibit Q = 5.96 ± 0.14 × 10^6^ with CE = 96.6 ± 1.0%, Q = 3.35 ± 0.03 × 10^6^ with CE = 99.1 ± 0.4% and Q = 2.15 ± 0.02 × 10^6^ with CE = 99.6 ± 0.6%, respectively. In Figure 8, a detailed view of resonance 4 fitted with a Lorentzian curve is reported. These results demonstrate that critical coupling can be achieved with our polymer waveguide, proving its effectiveness as an ideal coupler [20,21]. For comparison, the same MSR was characterized using a conventional fiber taper, achieving a similar maximum Q = 1.49 ± 0.07 × 10^7^ and CE = 99.0 ± 0.9%, as shown for the resonance in the inset of Figure 7.

### 3.2. Microbubble Resonator

**Figure 9:**
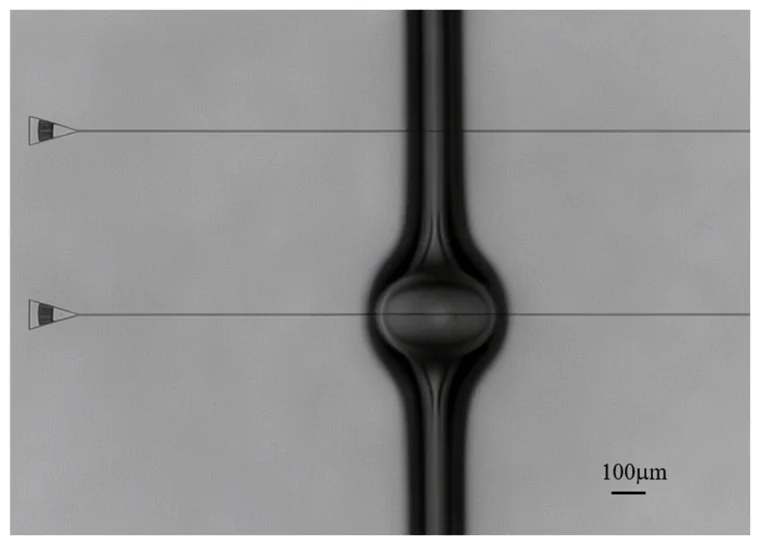
Microscope image of a silica microbubble positioned on the platform chip with an array of polymer waveguides.

The performance of the coupling system with microbubble resonators was also evaluated. MBRs attract significant attention due to their high Q, small mode volume, and extensive accordable resonance spectrum. Furthermore, MBRs are very promising in sensing applications because of their intrinsic microfluidic channel [22]. A picture of the MBR positioned over the platform is reported in Figure 9.

**Figure 10:**
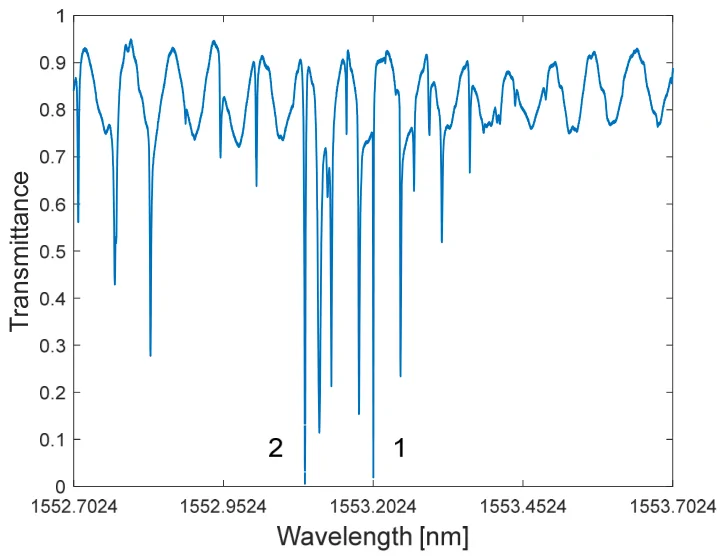
Transmission spectrum of the polymeric platform coupled to a silica microbubble resonator with a diameter of 375 µm.

**Figure 11:**
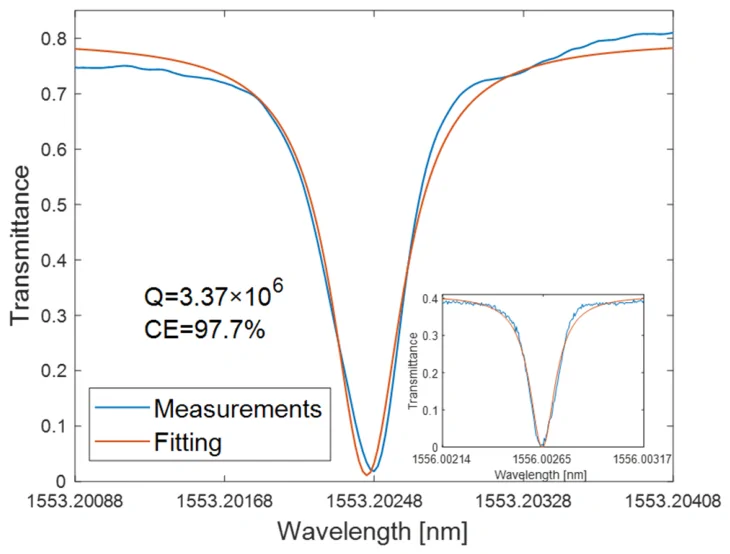
WGM resonance (n.1 in Figure 10) and its Lorentzian fit around λ = 1553.20 nm. The inset shows a similar WGM resonance measured with a fiber taper.

**Figure 12:**
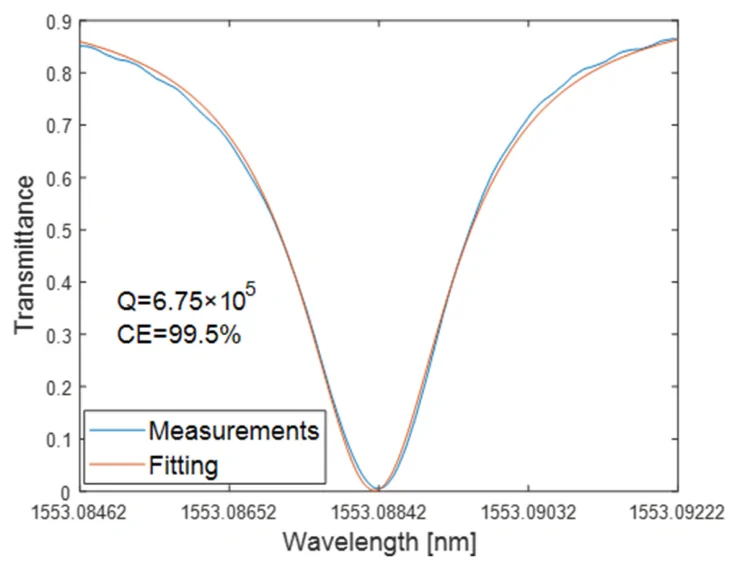
WGM resonance (n.2 in Figure 10) and its Lorentzian fit around λ = 1553.08 nm.

The transmission spectrum of the polymer platform coupled to a 375 µm diameter MBR is depicted in Figure 10. The maximum quality factor measured around λ = 1553.20 nm is Q = 3.37 ± 0.07 × 10^6^ with CE = 97.7 ± 1% (resonance 1). Figure 11 shows a detailed view of this resonance fitted with a Lorentzian curve. Critical coupling is achieved at resonance 2 with CE = 99.5 ± 0.2% and Q = 6.75 ± 0.03 × 10^5^ (Figure 12). In this case as well, the results are comparable with those achieved with fiber taper coupling (Q = 6.3 ± 0.07 × 10^6^, CE = 99.0 ± 0.7%), as shown in the resonance in the inset of Figure 11.

## 4. Discussion

The experimental results reported in the previous section demonstrate that high coupling efficiency can be achieved with polymer waveguides fabricated using polymers commonly employed in integrated optics like SU8 and PDMS. Furthermore, the integration of the waveguides with a grating coupler is demonstrated, thus bypassing the spatial constraints of edge-coupling (including cleaving and edge polishing) and enabling full utilization of the chip area for efficient device integration. Although the present fiber-to-waveguide coupling efficiency is limited—primarily due to the resolution constraints of the laser direct-write lithography system—literature results show that this can be substantially improved without introducing additional fabrication complexity [23].

In comparison with previous coupling platforms employing integrated optical waveguides based on total internal reflection and fabricated with polymers (Q = 3.4 × 10^6^, CE = 91%) [7] or dielectric materials (Q ≈ 10^4^, CE ≈ 6.5% [8]) ((Q ≈ 6 × 10^4^) [9]), the proposed approach achieves better results both in terms of quality factor and CE (Q = 5.96 × 10^6^, CE = 96.6%). Furthermore, polymer-based couplers reported in the literature are based on low refractive index fluorinated polymers, and the integration of fluorinated polymers into planar chips presents several fabrication challenges both related to their physicochemical properties and to the compatibility with standard microfabrication techniques. Compared with advanced geometrically engineered silicon waveguides such as SPARROW (CE = 95%, Q = 5 × 10^7^ with a microsphere diameter of 220 µm and Q~10^6^ and CE = 98.6% with a diameter of 274 µm [10]) and photonic crystal (Q = 1.34 × 10^6^, CE = 99.5% [11]), our platform delivers comparable performance but with the advantage of avoiding complex fabrication processes. Metamaterial-based structures achieve the best results obtained in the literature (CE = 99%, Q = 2 × 10^7^ [12]); however, the smaller size scale and the fabrication tolerances place stringent demands on the manufacturing approach that usually requires cutting-edge lithography like electron-beam lithography.

Although silica fiber tapers still provide ideal coupling performance for silica high-Q resonators, their micrometer-scale dimensions render them inherently fragile and highly susceptible to dust and moisture contamination, which degrades transmission over time, requiring complex packaging [24]. Embedding tapers in low-index polymers can simplify the packaging and mitigate these stability issues [25,26], but doing so alters the resonator’s intrinsic characteristics and restricts its application space, especially with respect to sensing applications. Furthermore, the constraints imposed by the fiber taper length result in packaged device sizes that are typically on the centimeter scale, with strong limitations on miniaturization and multiplexing. In contrast, waveguide coupling offers inherently superior robustness and integration at a microscale by eliminating these limitations. Specifically, planar integration with waveguides is the most promising strategy for transitioning whispering gallery mode resonators (WGMRs) from fragile laboratory prototypes to robust, practical devices [5]. From this perspective, the developed platform enables coupling performance comparable to a tapered optical fiber coupler, with all the advantages of planar technology allowing a simple and effective packaging of silica microresonators [8,9,15].

The proposed approach also opens the possibility to simply implement multiple add-drop configurations by coupling multiple waveguides to a single microresonator. The ability to precisely control, through the lithographic process, the distance between two or more waveguides will enable the development of enhanced optical devices exploiting phenomena such as all-optical signal processing and resonant tunneling [27,28].

## 5. Conclusions

In summary, a polymeric platform based on planar technology capable of ensuring efficient light coupling up to 99% to high intrinsic Q (up to 10^7^) silica WGM resonators was demonstrated. The proposed approach provides a reliable and high-performance coupling method via a simple fabrication process. The results show that critical coupling can be achieved with both high-Q MSRs and MBRs, as with fiber taper couplers, but will enable an easy integration of silica resonators into photonic chips.

## Figures and Tables

**Table 1:** Measured refractive indices of polymer waveguide materials.

Wavelength (nm)	n_SU8_	n_PDMS_
632.8	1.5912 ± 0.0003	1.4147 ± 0.0012
1550	1.5752 ± 0.0007	1.4072 ± 0.0026

## Data Availability

Data underlying the results presented in this paper are not publicly available at this time but may be obtained from the authors upon reasonable request.

## Abbreviations

The following abbreviations are used in this manuscript:

Q	Quality factor
CE	Coupling efficiency
WG	Waveguide
WGM	Whispering gallery modes
PDMS	Polydimethylsiloxane
SPARROW	Stripline pedestal anti-resonant reflecting optical waveguide
FEM	Finite element method
UV	Ultraviolet
PGMEA	Propylene-glycol-monoether-acetate
MSR	Microsphere resonators
MBR	Microbubble resonators

## References

[1] Ward J., Benson O. (2011). WGM microresonators: Sensing, lasing and fundamental optics with microspheres. Laser Photon. Rev..

[2] Strekalov D.V., Marquardt C., Matsko A.B., Schwefel H.G., Leuchs G. (2016). Nonlinear and quantum optics with whispering gallery resonators. J. Opt..

[3] Yu D., Humar M., Meserve K., Bailey R.C., Chormaic S.N., Vollmer F. (2012). Whispering-gallery-mode sensors for biological and physical sensing. Nat. Rev. Methods Primers.

[4] Braginsky V.B., Gorodetsky M.L., Ilchenko V.S. (1989). Quality-factor and nonlinear properties of optical whispering-gallery modes. Phys. Lett. A.

[5] Berneschi S., Farnesi D., Frigenti G., Pelli S., Conti G.N., Soria S. (2025). From laboratory to prototype: The last-mile issue in whispering gallery mode resonator-based devices. Opt. Mat..

[6] Knight J.C., Cheung G., Jacques F., Birks T.A. (1997). Phase-matched excitation of whispering-gallery-mode resonances by a fiber taper. Opt. Lett..

[7] Le T., Savchenkov A.A., Tazawa H., Steier W.H., Maleki L. (2006). Polymer optical waveguide vertically coupled to high-Q whispering gallery resonators. IEEE Photon. Technol. Lett..

[8] Amorim V.A., Frigenti G., Baldini F., Berneschi S., Farnesi D., Jorge P.A., Maia J.M., Nunzi Conti G., Santos PSSdos Marques P.V. (2024). Integrated all-in-silica optofluidic platform based on microbubble resonator and femtosecond laser written surface waveguide. IEEE Sens. J..

[9] Zamora V., Preussler P., Herter J., Marx S., Schröder H., Schneider-Ramelow M. (2023). On-chip integration of optical microbottles for biosensing. Frontiers in Biological Detection: From Nanosensors to Systems XV.

[10] Laine J.-P., Little B.E., Lim D.R., Tapalian H.C., Kimerling L.C., Haus H.A. (2000). Microsphere resonator mode characterization by pedestal anti-resonant reflecting waveguide coupler. IEEE Photon. Technol. Lett..

[11] Zhuang Y., Kumazaki H., Fujii S., Imamura R., Daud N.A.B., Ishida R., Chen H., Tanabe T. (2019). Coupling of a whispering gallery mode to a silicon chip with photonic crystal. Opt. Lett..

[12] Farnesi D., Pelli S., Soria S., Nunzi Conti G., Le Roux X., Montesinos Ballester M., Vivien L., Cheben P., Alonso-Ramos C. (2021). Metamaterial engineered silicon photonic coupler for whispering gallery mode microsphere and disk resonators. Optica.

[13] Li H., Wang Y., Lin Z., Zhang S., Zhang Z., Zhang C., Tang C., Li E., Prades J.D. (2023). Design, fabrication and characterization of arrayed waveguide grating devices based on different polymers. Opt. Comm..

[14] Grimaldi I.A., Testa G., Bernini R. (2015). Flow through ring resonator sensing platform. RSC Adv..

[15] Persichetti G., Grimaldi I.A., Testa G., Bernini R. (2018). Self-assembling and packaging of microbottle resonators for all-polymer lab-on-chip platform. Sens. Actuator A.

[16] Ma H., Jen A.K.-Y., Dalton L.R. (2002). Polymer-Based Optical Waveguides: Materials, Processing, and Devices. Adv. Mater..

[17] Vermeulen D., Van Acoleyen K., Ghosh S., De Cort W., Yebo N.A., Hallynck E., De Vos K., Debackere P.P.P., Dumon P., Bogaerts W. Efficient tapering to the fundamental Quasi-TM mode in asymmetrical waveguides. In Proceedings of European Conference on Integrated Optics.

[18] Berneschi S., Farnesi D., Cosi F., Conti G.N., Pelli S., Righini G.C., Soria S. (2011). High Q silica microbubble resonators fabricated by arc discharge. Opt. Lett..

[19] Korček R., Medina Quiroz D., Wilmart Q., Edmond S., Cheben P., Vivien L., Alonso-Ramos C., Benedikovič D. (2023). Library of single-etch silicon nitride grating couplers for low-loss and fabrication-robust fiber-chip interconnection. Sci. Rep..

[20] Gorodetsky M.L., Ilchenko V.S. (1999). Optical microsphere resonators: Optimal coupling to high-Q whispering-gallery modes. J. Opt. Soc. Am. B.

[21] Spillane S.M., Kippenberg T.J., Painter O.J., Vahala K.J. (2003). Ideality in a fiber-taper-coupled microresonator system for application to cavity quantum electrodynamics. Phys. Rev. Lett..

[22] Testa G., Persichetti G., Bernini R. (2024). Optofluidic biosensing: Devices, strategies, and applications. TrAC-Trends Anal. Chem..

[23] Waldhäusl R., Schnabel B., Dannberg P., Kley E.B., Bräuer A., Karthe W. (1997). Efficient coupling into polymer waveguides by gratings. Appl. Opt..

[24] Kang Z., Chen T., Zhang J., Yang B., Tang D., Wang K., Yang Y. (2024). Packaging of WGM resonator coupled with tapered fiber for various application scenarios. Opt. Laser Technol..

[25] Yan Y.Z., Zou C.L., Yan S.B., Sun F.W., Ji Z., Liu J., Zhang Y.G., Wang L., Xue C.Y., Zhang W.D. (2011). Packaged silica microsphere-taper coupling system for robust thermal sensing application. Opt. Express.

[26] Tang T., Wu X., Liu L., Xu L. (2016). Packaged optofluidic microbubble resonators for optical sensing. Appl. Opt..

[27] Pöllinger M., Rauschenbeutel A. (2010). All-optical signal processing at ultra-low powers in bottle microresonators using the Kerr effect. Opt. Express.

[28] Crespo-Ballesteros M., Yang Y., Toropov N., Sumetsky M. (2019). Four-port SNAP microresonator device. Opt. Lett..

